# Outcomes of Enhanced Recovery After Surgery (ERAS) Protocol Implementation in Major Gynecologic Procedures: A Prospective Case-Control Study

**DOI:** 10.7759/cureus.84335

**Published:** 2025-05-18

**Authors:** M Radha, Kasturi Donimath, H Harshitha, Madhuri Kurdi, Kaushik Theerth

**Affiliations:** 1 Obstetrics and Gynaecology, Bala Gangadharanatha Swamiji (BGS) Global Institute of Medical Sciences, Bengaluru, IND; 2 Obstetrics and Gynaecology, Tatwadarsha Hospital, Hubballi, IND; 3 Anaesthesiology, Karnataka Medical College and Research Institute, Hubballi, IND; 4 Anaesthesiology, Medical Trust Hospital, Ernakulum, IND

**Keywords:** enhanced recovery after surgery (eras) protocols, length of hospital stay, major gynecological surgery, paralytic ileus, post-op complications, post-op pain management

## Abstract

Background and objectives

Enhanced recovery after surgery (ERAS) protocols have shown promise in improving patient outcomes across various surgical specialties. However, their adoption in gynecologic surgery has been relatively slow. This study aimed to evaluate the effectiveness of an ERAS protocol specifically tailored for major gynecologic procedures.

Methods

Following the Ethical Committee’s approval and patient consent, a prospective case-control study was conducted over 18 months. The study included 180 women undergoing major gynecologic surgery, divided into Pre-ERAS (n=90) and ERAS (n=90) groups. The ERAS protocol incorporated preoperative education, carbohydrate loading, minimal bowel preparation, standardized anesthesia and fluid management, early mobilization, early feeding, and multimodal pain management, while Pre-ERAS patients received conventional treatment as per institutional protocol.

Results

ERAS patients experienced a faster return of bowel function, with eight patients (8.9%) having the first bowel sounds within four hours. All ERAS patients ambulated within 24 hours, compared to 52 patients (57.8%) in the Pre-ERAS group. Pain management was superior in the ERAS group, with 70 patients (77.8%) reporting a pain score of 3 on day three, versus 24 patients (26.7%) in the Pre-ERAS group. Postoperative complications were significantly reduced in the ERAS group (n=12; 13.3% vs n=28; 31.1%). Notably, 73 ERAS patients (81.1%) were discharged within seven days, compared to 35 patients (38.9%) in the Pre-ERAS group.

Conclusion

The study demonstrates that implementing an ERAS protocol in major gynecologic surgery leads to faster recovery, reduced postoperative complications, and shorter hospital stays.

## Introduction

Enhanced recovery after surgery (ERAS) protocols represent a significant advancement in perioperative care for major gynecologic surgeries. These evidence-based, multimodal approaches aim to improve patient outcomes, reduce complications, and shorten hospital stays [1,2]. Gynecologic conditions requiring surgical intervention affect millions of women globally, with substantial impacts on quality of life and healthcare systems [3]. In the United States alone, approximately 600,000 hysterectomies are performed annually [4]. Worldwide, about 1,540,000 women underwent hysterectomy in 2016, according to the World Health Organization (WHO) data [5].

Traditional perioperative care in gynecologic surgery often involves prolonged fasting, liberal opioid use, and extended recovery periods, potentially leading to increased complications and prolonged hospital stays [6]. In contrast, ERAS protocols incorporate a range of interventions throughout the perioperative period, including preoperative patient education, carbohydrate loading, minimally invasive techniques, multimodal pain management, early mobilization, and early oral feeding [7].

While ERAS protocols have been successfully implemented in various surgical specialties, their adoption in gynecologic surgery has been relatively slower and less uniform [8]. This study aimed to evaluate the implementation and effectiveness of an ERAS protocol specifically tailored for major gynecologic surgery. We hypothesized that patients undergoing major gynecologic procedures with ERAS protocols will experience improved recovery, reduced complication rates, and shorter hospital stays compared to those receiving traditional perioperative care.

## Materials and methods

This prospective case-control study was conducted over 18 months (August 2022 to February 2023) after obtaining approval from the Ethical Committee of Karnataka Institute of Medical Sciences, Hubballi, India (approval no. KIMS:ETHICS COMM:318:2022-23; dated July 26, 2022).

A total of 180 women with American Society of Anesthesiologists (ASA) physical status I and II aged 18-65 years undergoing major gynecological surgeries such as total abdominal hysterectomy, non-descent vaginal hysterectomy, vaginal hysterectomy, Mayoward’s procedure, or myomectomy under regional anesthesia were assessed for fitness for surgery and included in the study while patients undergoing surgery for gynecologic oncology, laparoscopic surgery, and surgeries under general anesthesia, patients with body mass index >35 kg/m^2^ were excluded as patients under these categories had more propensity for prolonged surgeries and post-operative complications.

**Figure 1:**
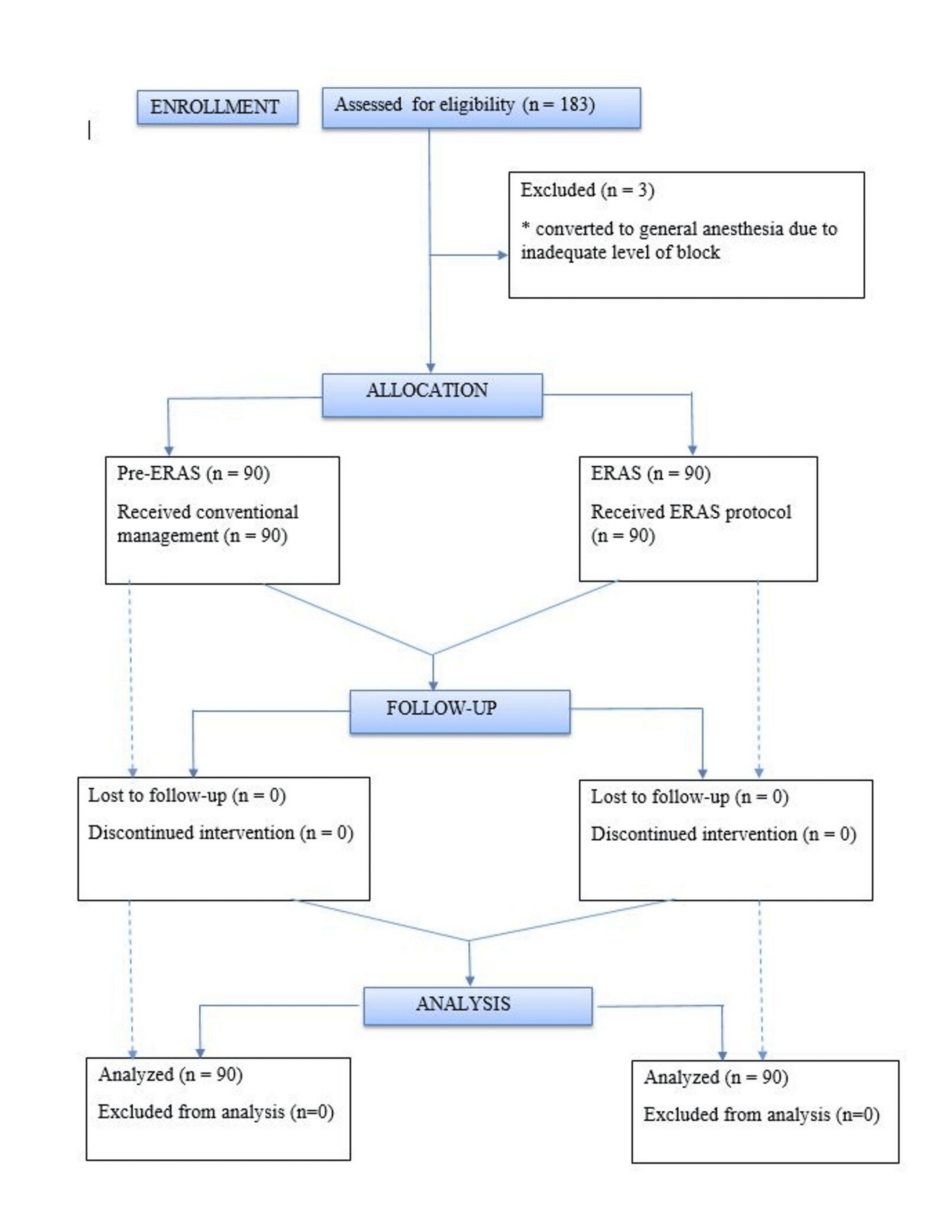
Consort diagram of present study

Taking into account the number of gynecological surgeries done in our institution on an annual basis, with a confidence limit of 95% and alpha error of 5%, power of study being 80% with precision of 5% and the percentage of patients who showed a positive result in the pilot article by Modesitt et.al. as n=50 (37%), the sample size was calculated using the formula $𝑛 &ge; (𝑍_{1- &alpha;/2} + Z_{1-&beta;})^{2} (\sigma_{1}^{2}+ \sigma_{2}^{2}\div r)\div (&mu;_{1}-&mu;_{2})^{2}$ to be 169 [9]. Considering the drop-outs, the sample size was rounded to 180, with 90 participants in each group. All patients who fit into the criteria were divided into two groups namely, Pre-ERAS (controls) (n=90) and ERAS (cases) (n=90) (Figure 1).

The Pre-ERAS group comprised patients admitted from August 1, 2022 to February 29, 2023 and they received conventional perioperative care. The ERAS group included patients admitted from June 1, 2023 to December 31, 2023 and they followed the ERAS protocol.

A detailed history of each patient was taken, including a detailed obstetric and menstrual history, medical history, and previous surgical history. A thorough physical examination of each subject was also done. Preoperative investigations, including routine hemogram and serum electrolytes, were done to exclude cases of anemia or any electrolyte imbalance.

Data collected included patient demographics, surgical details, postoperative recovery metrics (first bowel sounds, flatus, and stool passage), pain scores, complications, and length of hospital stay.

All subjects in the Pre-ERAS group were instructed to have nothing by mouth after midnight. Bowel preparation included Bisacodyl, two tablets (10 mg) and sodium phosphate enema the day before surgery, prophylactic antibiotic included cephalosporins administered one hour before surgery. All patients were operated under regional anesthesia. The duration of the surgery was found to be 1.5 to 2 hours, amount of blood loss was between 300 and 400 ml and any surgical complications were recorded.

Post-operatively, intravenous antibiotics were continued, which included a third-generation Cephalosporin and Metronidazole for two days followed by a switch to oral antibiotics from post-operative day three. Pain was assessed using the Numerical Pain Rating Scale (NPRS). Non-opioid analgesics such as Acetaminophen 1 g intravenous SOS and Diclofenac 75 mg intravenous SOS were used for moderate pain management (NRPS 5-7). Additional opioids such as Fentanyl (2 μg/kg) were used in case of severe pain (NPRS>8). No prokinetic agents were used.

The removal of urinary catheter was planned after 24 hours in total abdominal hysterectomy, after 48 hours in vaginal hysterectomy, and after 72 hours in Mayoward's procedure. Intestinal sounds were checked hourly with a stethoscope over the abdomen to note down the first appearance of bowel sounds. The first-time passing of flatus and the first evacuation time were also recorded by asking the patient. Other complications such as nausea, vomiting, bloating, and abdominal distention were noted. Post-operatively, complications such as fever, paralytic ileus, surgical site infection, urinary tract infections, and need for blood transfusion were noted.

Patients enrolled in the ERAS group followed the prescribed protocol (Table 1).

**Table 1:** Enhanced Recovery After Surgery (ERAS) Protocol followed in our study IV: intravenous, PO: per oral, TID: three times daily, QID: four times a day, POD: post-op day

ERAS Component	Intervention
Preoperative education	The patient receives one-on-one teaching about the ERAS pathway, her role in recovery, and expectations; Expectations set for the expected date of discharge
Carbohydrate loading drink	Glucose 100 g in 200 ml water given two hours before surgery
Bowel preparation	Bisacodyl two tablets (10 mg) the day before surgery
Antibiotics	Injection Ceftriaxone 1 g IV 60 minutes before skin incision
Anesthesia	Sub-arachnoid + Epidural anesthesia
Fluid management	Using Holliday-Segar method [10]
Multimodal pain management	Acetaminophen 1g IV TID followed by 500 mg PO QID from post-op day one; Epidural infusion with 0.125% Bupivacaine titrated to effect continued for 24-48 hours
Stimulation of gut motility	One hour after the surgery, patients were given a piece of artificially sweetened chewing gum, weighing around 2.4 g. They were asked to chew on the gum for 20 minutes
Early mobility	Out of bed and in chair on night of surgery; out of bed to walk at least three times each postoperative day
Early feeding	Allowed orally six hours after surgery; soft diet on POD1 and transitions to general diet as tolerated
Discharge criteria	Tolerating diet, ambulatory and pain well controlled on oral analgesia

Statistical analysis

Statistical analysis was performed using IBM SPSS Statistics for Windows, version 20.0 (IBM Corp., Armonk, NY) with chi-square tests and independent t-tests used to compare outcomes between the two groups. A p-value of ≤0.05 was considered statistically significant.

## Results

The demographic and clinical characteristics were similar between the two groups, ensuring comparability. The mean age, presenting complaints, and diagnoses were not significantly different. However, there was a notable difference in the distribution of surgical procedures, with the ERAS group having a higher proportion of total abdominal hysterectomy and bilateral salpingo-oophorectomy (TAH+BSO) procedures (n=26; 28.9% vs n= 12;13.3%) in the Pre-ERAS group (Table 2).

**Table 2:** Comparison of age and clinical data between the study groups n= number of participants, %= frequency AUB: abnormal uterine bleeding; PID: pelvic inflammatory disease; NDVH: non-descent vaginal hysterectomy; TAH: total abdominal hysterectomy; TAH+BSO: total abdominal hysterectomy and bilateral salpingo-oophorectomy Parameters were compared using chi-square test and a p-value ≤0.05 was considered to be significant.

Variable		Pre-ERAS	ERAS	Chi-square	p-value
Age (mean ± standard deviation) years		45.91 ± 7.19	46.53 ± 7.91	1.322	0.857
Complaints, n (%)	Heavy menstrual bleeding	56 (62.2%)	56 (62.2%)	0.755	0.86
	Mass per abdomen	01 (1.1%)	01 (1.1%)		
	Mass per vagina	20 (22.2%)	17 (18.9%)		
	Pain abdomen	13 (14.4%)	16 (17.8%)		
Diagnosis, n (%)	AUB	57 (63.3%)	64 (71.1%)	2.409	0.661
	Chronic PID	02 (2.2%)	00		
	Fibroid uterus	08 (8.8%)	09 (10%)		
	Prolapse uterus (Grade 2, 3)	22 (24.4%)	14 (15.6%)		
	Ovarian cyst	01 (1.1%)	03 (3.3%)		
Procedure, n (%)	Mayoward's	20 (22.2%)	17 (18.9%)	10.739	0.05
	Myomectomy	02 (2.2%)	04 (4.4%)		
	NDVH	29(32.2%)	27 (30%)		
	TAH	27 (30%)	16 (17.8%)		
	TAH+BSO	12 (13.3%)	26 (28.9%)		

Significant improvements in postoperative recovery were noted with the ERAS protocol. Early return of bowel function was more common in the ERAS group, with eight patients (8.9%) experiencing first bowel sounds within four hours compared to none in the pre-ERAS group. All ERAS patients ambulated within 24 hours, compared to only 52 patients (57.8%) in the Pre-ERAS group. Urinary catheter removal at 24 hours was more frequent in the ERAS group (n=58 (64.4%) vs n=36( 40%)). Pain management was also superior in the ERAS group, with 70 patients (77.8%) reporting a pain score of 3 on day three, compared to only 24 patients (26.7%) in the Pre-ERAS group (Table 3).

**Table 3:** Comparison of postoperative recovery of study participants n= number of participants, %= frequency Groups were compared using chi-square test and a p-value ≤0.05 was considered significant.

Characteristic	Pre-ERAS, n (%)	ERAS, n (%)	Chi-square	p-value
First bowel sounds <4 hours	0	8 (8.9%)	7.09	0.02
Ambulation <24 hours	52 (57.8%)	90 (100%)	45.667	<0.001
Urinary catheter removal at 24 hours	36 (40%)	58 (64.4%)	11.131	0.003
Pain score 3 on Day 3	24 (26.7%)	70 (77.8%)	52.641	<0.001

The ERAS group had a higher proportion of patients with no post-op complications (n=78, 86.7%). Notably, there were no cases of paralytic ileus in the ERAS group compared to six patients (6.7%) in the Pre-ERAS group. Fever and urinary complaints were also less frequent in the ERAS group (Table 4).

**Table 4:** Comparison of postoperative complications n= number of participants, %= frequency Groups were compared using chi-square test and a p-value ≤0.05 was considered to be significant.

Complications	Pre-ERAS, n (%)	ERAS, n (%)	Chi-square	p-value
No complications	62 (68.9%)	78 (86.7%)	11.829	0.018
Need for blood transfusion	07 (7.8%)	06 (6.7%)		
Fever	06 (6.7%)	02 (2.2%)		
Paralytic Ileus	06 (6.7%)	00		
Urinary complaints	09 (10%)	04 (4.4%)		
Total	90 (100%)	90 (100%)		

A significantly higher proportion of ERAS patients had a hospital stay of less than seven days (n=73, 81.1% vs n=35, 38.9%). The interval between surgery and discharge was also shorter in the ERAS group, with 64 patients (71.1%) discharged on day four compared to only two patients (2.2%) in the Pre-ERAS group (Table 5).

**Table 5:** Postoperative outcomes between the groups n= number of participants, % = frequency Outcomes were compared with chi-square test and a p-value of ≤0.05 was considered significant.

Outcome		PRE-ERAS [n (%)]	ERAS [n (%)]	Chi-square	p-value
Hospital stay	<7 days	35 (38.9%)	73 (81.1%)	31.45	<0.001
	8-14 days	53 (58.9%)	16 (17.8%)		
	>14 days	2 (2.2%)	1 (1.1%)		
Interval between surgery and discharge	3 days	0	1 (1.1%)	89.31	<0.001
	4 days	2 (2.2%)	64 (71.1%)		
	5 days	86 (95.6%)	25 (27.8%)		
	6 days	2 (2.2%)	0		

The implementation of the ERAS protocol in a major gynecologic surgery led to faster recovery, reduced postoperative complications, shorter hospital stays, and earlier discharge. These improvements were statistically significant across multiple parameters. The ERAS protocol appears to be highly effective in enhancing patient outcomes and potentially reducing healthcare costs associated with prolonged hospitalizations and complications.

## Discussion

This study demonstrates the significant benefits of implementing an ERAS protocol in major gynecologic surgery. The ERAS group showed faster recovery of bowel function, earlier ambulation, and earlier removal of urinary catheters compared to the Pre-ERAS group. These findings are consistent with other ERAS studies in gynecological surgery, such as Charoenkwan et al., who reported earlier return of bowel function and faster mobilization in their ERAS cohort [11].

Postoperative pain management was significantly improved in the ERAS group, with lower pain scores reported across all three postoperative days. This aligns with the findings of Dickson et al., who reported improved pain control with ERAS protocols in gynecological oncology surgery [12]. The reduced pain scores likely reflect the use of multimodal analgesia and opioid-sparing techniques, which are key components of ERAS protocols.

The ERAS group demonstrated lower overall complication rates compared to the pre-ERAS group (n=12, 13.3% vs n= 28, 31.1%). This is consistent with meta-analyses of ERAS in gynecological surgery, such as de Groot et al., who reported reduced complication rates with ERAS implementation in their systematic review [13]. The reduced complications likely reflect the cumulative benefits of early mobilization, optimized fluid management, and multimodal analgesia inherent in ERAS protocols [14-16].

Perhaps most significantly, the ERAS group had substantially shorter hospital stays, with 73 patients (81.1%) discharged within seven days compared to only 35 patients (38.9%) in the Pre-ERAS group. This aligns with numerous other studies, including Nelson et al. [8] who reported a median reduction in length of stay of two days with ERAS implementation in gynecological oncology. Our findings further support the efficacy of ERAS in reducing hospital stay without compromising patient safety, potentially leading to improved patient satisfaction and reduced healthcare costs.

The application of ERAS protocol for surgeries under regional anesthesia helped in keeping opioid use to a minimum. However, the exclusion of laparoscopic surgeries, oncological surgeries, and major surgeries under general anesthesia from the study limited our experience of outcomes in those scenarios. We anticipate further studies on them will have positive outcomes and help improve patient care.

## Conclusions

The ERAS protocol significantly enhances postoperative recovery in women undergoing major gynecologic surgery compared to traditional care. These findings support the adoption of ERAS protocols to improve clinical outcomes and patient recovery in gynecologic surgeries. Further research is recommended to validate these results across larger and more diverse populations.
